# Value of information in natural resource management: technical developments and application to pink-footed geese

**DOI:** 10.1002/ece3.1363

**Published:** 2015-01-04

**Authors:** Byron K Williams, Fred A Johnson

**Affiliations:** 1 The Wildlife Society5410 Grosvenor Lane, Suite 200, Bethesda, Maryland, 20814-2144; 2 Southeast Ecological Science Center, U.S. Geological Survey7920 NW 71 Street, Gainesville, Florida, 32653

**Keywords:** Adaptive management, conservation, decision making, natural resource management, pink-footed geese, uncertainty, value of information

## Abstract

The “value of information” (VOI) is a generic term for the increase in value resulting from better information to guide management, or alternatively, the value foregone under uncertainty about the impacts of management (Yokota and Thompson, *Medical Decision Making* 2004; **24**: 287). The value of information can be characterized in terms of several metrics, including the expected value of perfect information and the expected value of partial information. We extend the technical framework for the value of information by further developing the relationship between value metrics for partial and perfect information and describing patterns of their performance. We use two different expressions for the expected value of partial information to highlight its relationship to the expected value of perfect information. We also develop the expected value of partial information for hierarchical uncertainties. We highlight patterns in the value of information for the Svalbard population of the pink-footed goose (*Anser brachyrhynchus*), a population that is subject to uncertainty in both reproduction and survival functions. The framework for valuing information is seen as having widespread potential in resource decision making, and serves as a motivation for resource monitoring, assessment, and collaboration.

## Introduction

The “value of information” (VOI) is a generic term for the increase in value resulting from better information to guide management. Alternatively, it can be viewed as value foregone under uncertainty about the impacts of management (Yokota and Thompson 2004). One of many applications might use the value of information to inform the assessment of monitoring effectiveness. For example, an increase in the value expected from reducing uncertainty can be compared against opportunity and other costs associated with collecting and analyzing information, to determine whether and how monitoring should be undertaken (Hauser et al. 2006; McDonald-Madden et al. 2010).

The concept of a value of information has been recognized for several decades and is now well developed. Raiffa and Schlaifer (1961) provided one of the first seminal treatments, coining the name and developing many of its key expressions. Since then it has been applied in economics, finance, medicine, engineering, and many other fields (e.g., Frauendorfer 1992; Bontems and Thomas 2000; Karnon 2002; Koerkamp et al. 2006; Eidsvik et al. 2008). With few exceptions (see, for example, Moore and McCarthy 2010; Williams et al. 2011; Smith et al. 2013; Johnson et al. 2014b), applications in ecology and natural resources management have involved an assessment of the utility of information with noniterative decision making (e.g., Runge et al. 2011, Johnson et al. 2014a). Williams et al. (2011) described a framework for VOI that is applicable for dynamic resource systems in which actions are taken periodically over an extended time frame. The framework includes sequential decision making over a timeframe, resources that change in response to environmental fluctuations and management decisions, and an accounting for uncertainty about the processes driving resource dynamics in response to environmental fluctuations and management decisions. Key components include a range of potential actions, models that forecast resource changes, measures of confidence in the models, and a valuation protocol to assess the effectiveness of management (Williams et al. 2011).

The concept of value can take many forms in natural resources. For example, it can be defined for exploited systems as the temporal sum of harvests over an extended time horizon. With imperiled species, value can be defined in terms of annual growth rate, or the probability of persistence, with a value of unity if the species persists in the future and zero if it does not. With invasive species, value might be represented by the number of habitat patches not infested, or the opportunity costs associated with infestation. These and other value elements can be incorporated into management objectives and used to guide decision making through, for example, multicriteria decision analysis (Keeney and Raiffa 1976).

In what follows, we expand on earlier work by the authors and colleagues (Williams et al. 2011; Johnson et al. 2014b) and consider several forms of the value of information and linkages among them. We discuss the drivers of patterns in the value metrics and illustrate the metrics for the Svalbard population of the pink-footed goose (*Anser brachyrhynchus*).

## Resource Management Under Uncertainty

To describe the value of information in terms of dynamic resource systems, a notation and a few key value expressions are needed. Thus, we denote the state of a resource system at a particular time by *x*, the system state in the subsequent time period by *x*′, and management action by *a*. The action taken at a particular time is seen as part of a policy *A*_*t*_ of state- and time-specific actions over a time frame starting at *t*. Letting *R*(*a*|*x*) represent an immediate return corresponding to action *a* when the system state is *x*, a value function

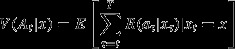
accumulates these returns over time for a particular policy *A*_*t*_ (Williams et al. 2002). The value function can be expressed recursively as

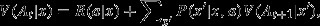
where *P*(*x*′|*x*, *a*) is the probability of transition from *x* to *x*′ given that action *a* is taken. An optimal policy and values can be identified with Bellman's equation (Puterman 1994; Bertsekas 1995):

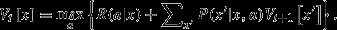
3 A key extension allows for uncertainty about resource processes, including the system transition probabilities. Here we use *R*_*k*_(*a*|*x*) and *P*_*k*_(*x*′|*x*, *a*) to denote returns and transitions that are based on a particular model *k* of resource dynamics. Uncertainty about the most appropriate model is given by a time-varying distribution *q* of model confidence measures called the model state, which evolves through time according to Bayes' theorem (Lee 1989):

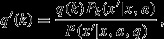
4

where the averaging over model probabilities *q*(*k*) in the model state produces

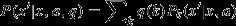
and

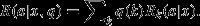
A value function incorporating process uncertainty is

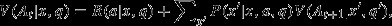
where

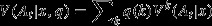
with

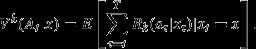


An optimal policy and values for this situation again can be determined by Bellman's equation (Williams et al. 2002):

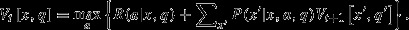
10 Note that the argument for the value function in Eq. (3) now includes both the system and model states.

## The Expected Value of Perfect Information

As described in Williams et al. (2011), the expected value of perfect information (*EVPI*) for a dynamic system with process uncertainty uses the optimal values in Eqs. (1) and (3):

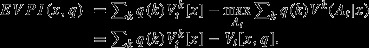
11
*EVPI* is essentially the average of value maxima minus the maximum of an average value over the models.

The term 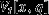 in Eq.  (4) is the optimal value produced by active adaptive optimization (Williams 2011), which in many circumstances can be difficult to identify. A good approximation is obtained with passive adaptive optimization, an approach that accounts explicitly for uncertainty but not learning in the optimization (Williams 2011; Williams and Johnson 2013). Because a passive approach typically produces somewhat lower values, its use in computing *EVPI* can produce values that are somewhat positively biased (see below).

### Application to pink-footed geese

The need for informed management of European goose populations is increasingly urgent because many populations have grown dramatically in recent decades (Madsen et al. 1999; Fox et al. 2010). Although geese are regarded as a valuable resource, the growth in numbers and their tendency to concentrate on farmlands have growth agricultural conflicts in wintering and staging areas. In some Arctic regions, increasing goose abundance has resulted in overexploitation of vegetation, causing long-term degradation of tundra habitats.

The Svalbard population of the pink-footed goose (*Anser brachyrhynchus*) was recently selected as the first test case in Europe for development of an international harvest management plan to help address these concerns. The Svalbard population breeds primarily in Spitsbergen, migrates through Norway, and winters primarily in Denmark, the Netherlands, and Belgium. Although these geese have been well studied, much uncertainty remains about population dynamics and the effects of harvest. Managers are currently considering nine alternative population models that are based on different combinations of three survival and three reproductive models (Johnson et al. 2014b). These models represent a wide range of possibilities concerning whether demographic rates are density dependent or independent, and whether spring temperatures on the breeding grounds affect vital rates.

The model-specific harvest management objective for this problem is

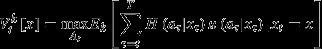
where *H* (*a*_*τ*_|*x*_*τ*_) is the amount of harvest in year *τ* and harvest utility is:

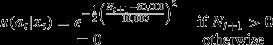
and *N*_*t*+1_ is total population size. The objective function seeks to maximize sustainable harvest, but devalues harvest decisions that are expected to result in a subsequent population size differing from the population goal, with the degree of devaluation increasing as the difference between population size and the goal increases.

**Figure 1:**
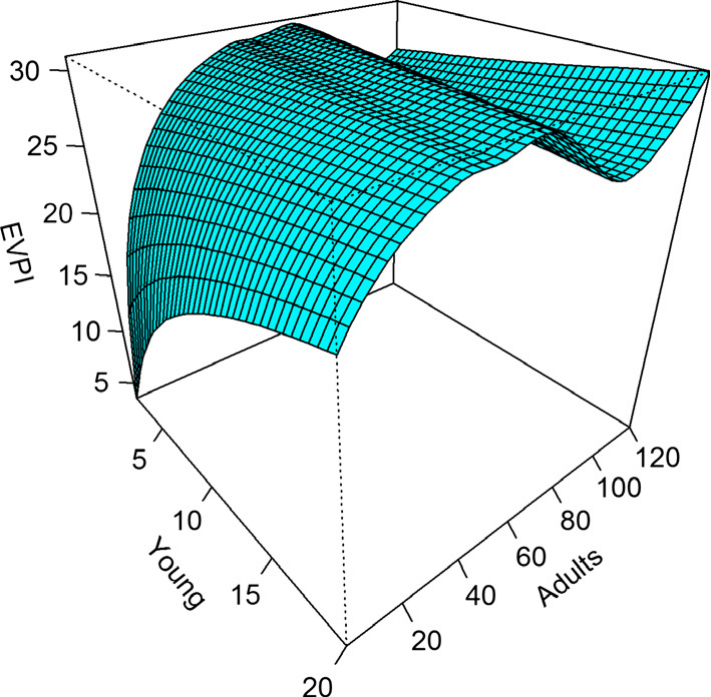
State-dependent values of the expected value of perfect information (*EVPI*) for nine equally weighted alternative models of pink-footed goose population dynamics, based on the number of young and adults (in thousands) in the population and conditioned on the average spring temperature. *EVPI* represents cumulative objective values (in thousands) over a 50-year time horizon, where the management objective is to maximize harvest and maintain a population size near 60 thousand.

Johnson et al. (2014b) calculated the expected value of perfect information for this problem by determining model-specific optimal strategies and averaging the corresponding optimal values, then subtracting the optimal values based on averaged transitions using passive adaptive optimization. In their case, they used time- and state-averaged values to suggest that *EVPI* for equal model weights represented only a 3.0% gain in objective value. Thus, the passive adaptive strategy was expected to perform relatively well, regardless of the most appropriate model of population dynamics. We also used their results to discern state-specific patterns in *EVPI* (Fig.1). We used cumulative objective values for a 50-year time horizon and examined how *EVPI* varied with the number of young and adults in the population, conditioned on the average spring temperature. Values of *EVPI* were lowest for small population sizes where all of the alternative models prescribe low harvest rates. Interestingly, values of *EVPI* were highest along a ridge representing combinations of adult and young abundances that produce a population size close to the management goal of 60 thousand. We can infer from this that it is within this range of states that the selection of the optimal model-specific policy is most ambiguous.

**Figure 2:**
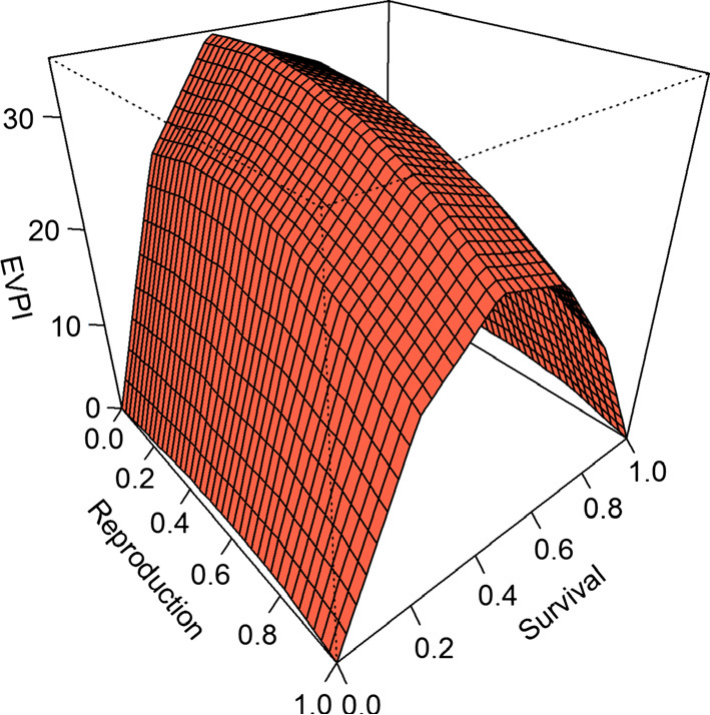
Model-dependent values of the expected value of perfect information (EVPI) for four alternative models of pink-footed goose population dynamics, conditioned on an intermediate number of young (10,000) and adults (60,000) in the population and the average spring temperature. The axes labeled “survival” and “reproduction” represent the probabilities that those demographic rates are density dependent.

We also investigated how *EVPI* varies with model state. We focused on only four of the nine alternative models for pink-footed geese, with all four using spring temperature as a predictor of both survival and reproduction. However, each alternative model expresses a different hypothesis about whether survival, reproduction, or both are density dependent (models M1, M2, M4, and M5 in Johnson et al. 2014b). As before, we used cumulative objective values for a 50-year time horizon and examined how *EVPI* varied with the probabilities that survival and reproduction are density dependent (Fig.2). We conditioned on an intermediate population size (10,000 young and 60,000 adults) and the average spring temperature. At the bounds of the probability space, *EVPI = 0* because there is no uncertainty. *EVPI* increases to a maximum in the interior of the probability space and is most strongly affected by the probability that survival is density dependent. Variation in the probability that reproduction is density dependent produces relatively small changes in *EVPI*.

## Expected Value of Partial Information

In many situations, multiple sources of uncertainty can be identified. An example is uncertainty in both reproduction and survival functions, with each contributing to overall model uncertainty. In such a situation, it is useful to identify which source of uncertainty has the larger impact on value, so as to help focus efforts to reduce or eliminate uncertainty. The expected value of partial information (*EVPXI*) addresses the loss of value for multiple uncertainty sources. Although *EVPXI* can in concept accommodate three or more uncertainty factors, to simplify notation we assume here that two sources are at play, with different functional forms representing each source.

Thus, assume that the models under consideration are indexed by *k*′ and *k*′′, where both indices are used to identify a particular model (*k*′, *k*′′). Either index alone identifies groups of models. For example, index *k*′ = 1 corresponds to the models {(1, 1), (1, 2), (1, 3), …}, whereas *k*′ = 2 corresponds to the models {(2, 1), (2, 2), (2, 3), …}. In this context, the uncertainty factors can be thought of as crossed, with each level of one factor paired with every level of the other. Because both indices are used to identify the models, there are two possible measures of *EVPXI*, which we denote by *EVPXI*^*K*′^(*x*, *q*) and *EVPXI*^*K*′′^(*x*, *q*). The superscript *K*′ is used here to connote information value for model sets that are defined as above by different levels of the model index *k*′. As with *EVPI*, both *EVPXI*^*K*′^ and *EVPXI*^*K*′′^ are conditional on system and model states.

Here we use the following notation in calculating *EVPXI*^*K*′^(*x*, *q*):

**Table:** 

(*k*′,*k*′′)	bivariate model designation
*q* = *q*_*k*′_*q*_*k*”|*k*′_ = *q*_*k*”_*q*_*k*′|*k*”_	model state, expressed in terms of conditional and marginal probabilities for *k*′ and *k*′′. Elements of *q* are probabilities 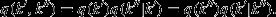
*V*^*k*′*k*”^(*A*_*t*_|*i*)	value of policy *A*_*t*_ for model (*k*′,*k*′′)

The calculation of *EVPXI*^*K*′^(*x*, *q*) is essentially a 3-step process:

**Step 1**: For a specific *k*′, average the value functions for models (*k*′, 1), (*k*′, 2), (*k*′, 3),… based on the conditional model state *q*_*k*′′|*k*′_:

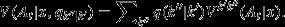
14

**Step 2**: Compute the maximum value for this average, that is,

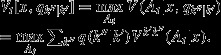
15

Do the computations in steps (1) and (2) for each value *k*′.

**Step 3**: Use the *V*(*A*_*t*_|*x*, *q*_*k*′′|*k*′_) values from Eq. (5) and *V*_*t*_[*x*, *q*_*k*′′|*k*′_] values from Eq. (6) to compute what is essentially *EVPI* in Eq. (4) based on these values:

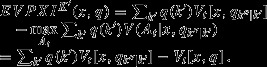
16

An analogous expression for *EVPXI*^*K*′′^(*x*, *q*) is obtained by switching *k*′ and *k*′′ in the above formulas.

### Alternative treatment of EVPXI

A simple manipulation of Eq.  7 provides an alternative method for calculating *EVPXI*, and some additional insight into its relationship to *EVPI*. Rewriting Eq. (7) by adding and subtracting the term 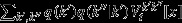 produces

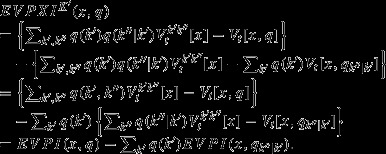
17

The expression 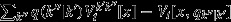 in Eq. (8) is simply the expected value of perfect information 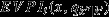 for the models (*k*′,*k*′′) that are grouped under the particular value *k*′. This provides another way to calculate *EVPXI*:

1. **Step 1**: Calculate *EVPI* (*x*, *q*)
2. **Step 2**: Calculate *EVPI* (*x*, *q*_*k*′′|*k*′_) for each *k*′
3. **Step 3**: Average the values in step 2 over *q*_*k*′_
4. **Step 4**: Subtract the average in step 3 from *EVPI* (*x*, *q*) in step 1

The result is *EVPXI*^*K*′^(*x*, *q*).

On reflection, Eq. (8) makes sense: The term *EVPI* (*x*, *q*_*k*′′|*k*′_) is simply the *EVPI* for all the models characterized by the index value *k*′, that is, models (*k*′,1), (*k*′,2), (*k*′,3), It therefore represents the loss of value because of uncertainty among models associated with index *k*′. The losses corresponding to each value of *k*′ are averaged over *q*_*k*′_, and that average is subtracted from the loss of value for the whole model set as represented by *EVPI* (*x*, *q*). The difference is the residual loss of value after one accounts as above for the loss of value from the uncertainty tied to index *k*′′. In that sense, the difference really is a value of partial information for the uncertainty index *k*′.

### Application to pink-footed geese

Johnson et al. (2014b) calculated the expected value of partial information for the pink-footed goose harvest management problem described above, focusing on the expected gain in management performance if uncertainty about either the survival or reproductive processes could be resolved. For this example, there are three alternative survival models and three reproductive models, and a value for *EVPXI* was calculated for each uncertainty factor. Using time- and state-averaged values, the authors found that the value accruing to elimination of uncertainty about the survival process was substantially higher (a gain of 2.2%) than the value associated with elimination of uncertainty about the reproductive process (a gain of 0.1%). This result is consistent with evidence that variation in survival is more important than variation in reproduction in regulating population growth of relatively long-lived avian species (Stahl and Oli 2006). The direct effect of the control variable (harvest) on survival also could have played a role in highlighting the importance of uncertainty about the survival process.

**Figure 3:**
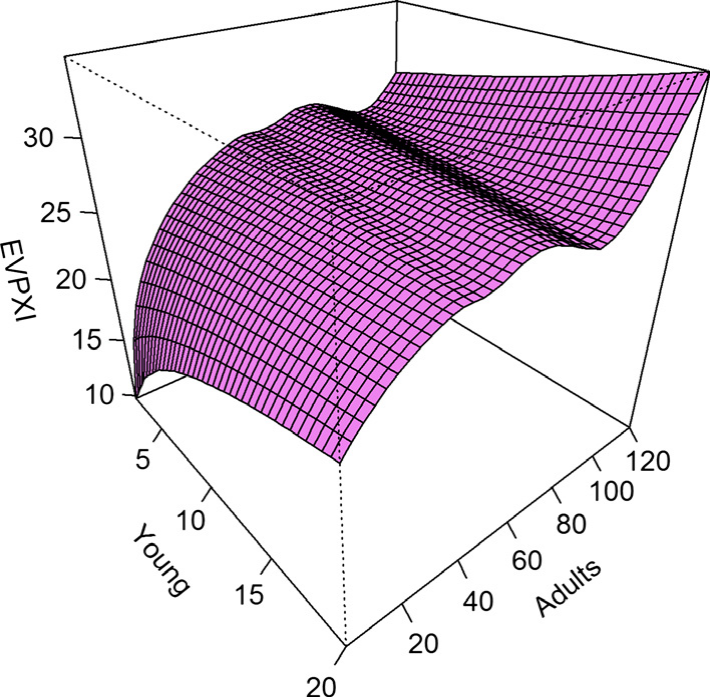
State-dependent values of the expected value of partial information (*EVPXI*) for three equally weighted alternative survival models of pink-footed goose population dynamics, based on the number of young and adults (in thousands) in the population and conditioned on the average spring temperature. *EVPXI* represents cumulative objective values (in thousands) over a 50-year time horizon, where the management objective is to maximize harvest and maintain a population size near 60 thousand.

We also used their results to discern state-specific patterns in *EVPXI* for the survival models (Fig.3). As before, we used cumulative objective values for a 50-year time horizon and examined how *EVPXI* varied for the number of young and adults in the population, conditioned on the average spring temperature. State-specific values of *EVPXI* generally increased with increasing population size, suggesting that it is at higher population levels that there is the greatest ambiguity about optimal harvest rates.

## Hierarchical Uncertainty Factors

In the above development, the models incorporate two forms of uncertainty, with different combinations of indices *k*′ and *k*′′ denoting the models under consideration. Here we consider a somewhat different uncertainty situation, in which the models are separated into two distinct groups according to some distinguishing feature. There is uncertainty about which group more effectively represents system dynamics, and the challenge is to select the appropriate group.

In this situation, the probability structure for model uncertainty is hierarchical rather than combinatorial, with *k*′ now denoting group identity and *k*′′ specifying a particular model within a group. As above, both indices are needed to identify an individual model in the complete set of candidate models. The hierarchical structure of uncertainty is captured in the conditional nature of the joint probability of these indices: *q*(*k*′, *k*′′) = *q*(*k*′)*q*(*k*′′|*k*′). The issue here is to assess the value of information in the grouping.

The expected value of partial information provides a natural approach for this situation. Thus, the residual value of information, obtained as in Eq. (8) by subtracting the average “within-group” value of information from the overall expected value of perfect information, produces a metric to help focus on the most appropriate group of models in reducing the loss of information from uncertainty. Thus, similar values for group-specific *EVPI*s can produce a small residual value in Eq. (8). In consequence, a low value for *EXPXI* suggests that all groups contribute to the loss of value from uncertainty, so one cannot safely focus on a specific group to reduce that loss. On the other hand, a large value for *EVPXI* can result from substantial differences among group-specific *EVPI*s, suggesting that the loss from uncertainty can be addressed by focusing on select groups with higher within-group *EVPI*s.

This framework allows one to consider the value of information for an individual model. To see how, let the models be divided into two groups, with one group consisting of a single model and the other consisting of the remaining models. Letting *k* = 2, …, *K* for the models in the larger group and *k *= 1 for the model in the singleton set, from Eq. (7) we have

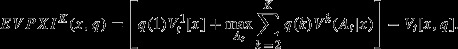
18

From this expression, *EVPXI* is seen to increase with an increasing value for model 1 relative to the other models.

### Application to pink-footed geese

**Figure 4:**
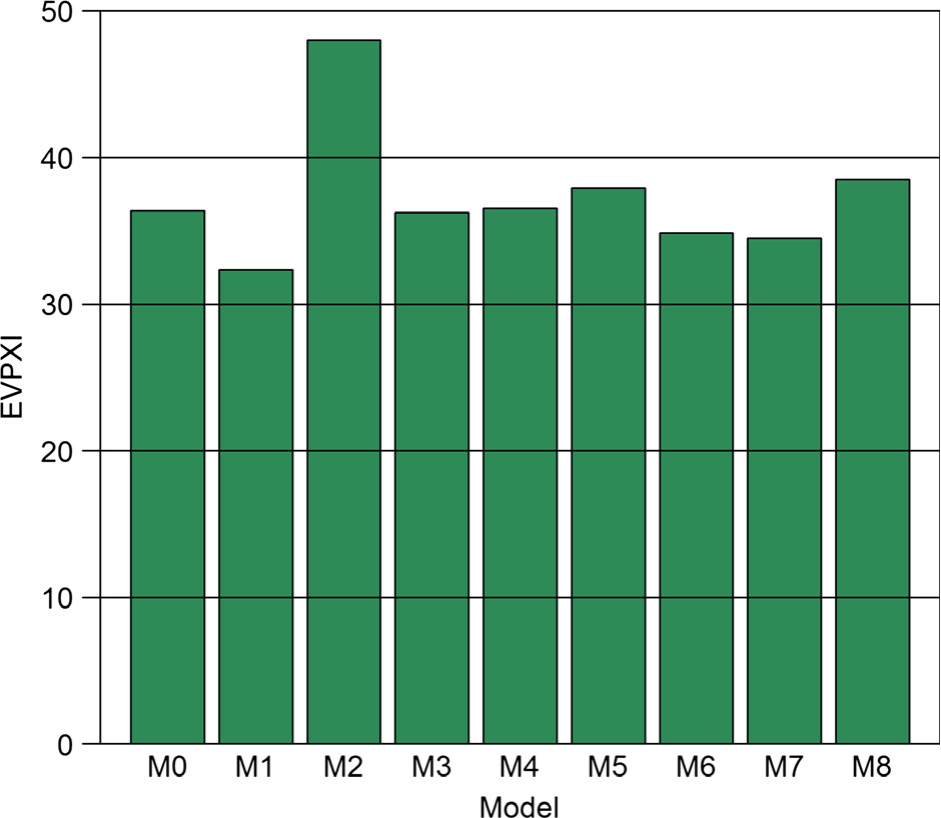
The value of eliminating uncertainty for each of the nine alternative models of pink-footed goose population dynamics (*EVPXI*), conditioned on an intermediate number of young (10,000) and adults (60,000) in the population and the average spring temperature, and with the nine models weighted equally.

We calculated *EVPXI* for each of the nine alternative models of pink-footed goose population dynamics from Johnson et al. (2014b) (Fig.4). As before, we used cumulative objective values for a 50-year time horizon and conditioned on an intermediate population size (10,000 young and 60,000 adults) and the average spring temperature. The *EVPXI* did not vary greatly among models, suggesting that one should not focus on any particular group of models to reduce the loss of value attendant to uncertainty. Model M2 had the highest *EVPXI*; this is the model in which both survival and reproduction depend on density and spring temperatures. For this model, the values of the two optima in Eq. (9), 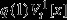 and 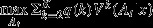, were dissimilar, resulting in a relatively large *EVPXI*. In other words, it is uncertainty about this model that results in the greatest loss of value.

## Discussion

Given the ever-present limitations on funding to conduct research, a key question that should be considered in natural resources science concerns the relative usefulness of investigations for improving management. The value of information addresses this question, by asking about the loss of value from uncertainty that could be reduced or eliminated by the investigations.

A formal description of the value of information builds on a value function and the loss in value that is attributable to uncertainty. Specifically, *EVPI* is described in terms of the added value for optimal decision making with the elimination of uncertainty, that is, the difference between potential valuation with the elimination of uncertainty and the highest value attainable in its presence:

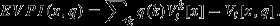


As mentioned above, the second term in this contrast is the value produced by active adaptive optimization.

It is of course possible to contrast optimal valuation and an associated value with any suboptimal strategy. One example is passive adaptive optimization, in which decision making is informed by system state but not the potential for learning (Williams 2011). Whereas active AM incorporates the potential for learning through management interventions, passive AM focuses on resource objectives, with learning a useful but unintended by-product of decision making (Walters 1986). Both forms of AM recognize uncertainty, both allow for uncertainty to be adjusted iteratively through time, and in both recognizing that valuation is conditional on the system and model states. But a key difference is the degree to which the objectives guiding decision making emphasize the reduction of uncertainty. As indicated above, the active adaptive form incorporates learning potential in the decision making process itself:

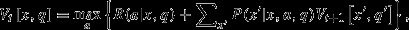
where learning is represented by the transition from model state *q* to state *q*′. On the other hand, a useful form of passive adaptive decision making effectively assumes the model state will remain unchanged for the remainder of the time frame:

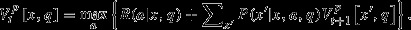


In this instance, decision making is not influenced by the potential for learning, even though learning does occur once the model state is updated with postdecision monitoring data. Here we denote valuation in passive decision making with a superscript *p* on the value terms. The values produced with passive adaptive management are almost always less than those for active adaptive management (Williams et al. 2002). It is this form of passive adaptive management that was used in the pink-footed goose example.

To illustrate the effect of suboptimal decision making, consider the use of 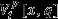 rather than 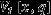 in the equation for *EVPI*. Then

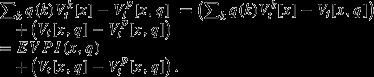


It is easy to see that suboptimal decision making increases the contrast between valuation under uncertainty and valuation with its elimination. In this particular case, the effect 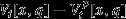 is often marginal, because passive optimal decision making typically approximates active decision making in its performance. However, the effect can be substantially greater with nonoptimal decision making, for example with stationary decisions or decision making that fails to account for changing system state through time. Under these circumstances, the metric represents the value of eliminating uncertainty in comparison with a decision making process that is less informed, although more frequently used, than the optimal decision making assumed in *EVPI*. One implication is that *EVPI* alone underrepresents the overall value of information in decision making.

As indicated above, the expected value of perfect information is the minimum loss in value that can be expected for a given model state (Yokota and Thompson 2004). The size of the loss depends on the amount of variation in model-specific values, with relatively small loss if the model values are similar. Under these conditions, there is little to be gained in determining which model best represents resource dynamics. In general, the magnitude of *EVPI* depends on the particular model state and ranges from 0 in the absence of uncertainty to a maximum at some point in the interior of the model state space. Small changes around a particular model state can be expected to produce linear changes in *EVPI* (Williams et al. 2011).

The situation for expected value of partial information is somewhat more complicated. In this case, there are multiple sources of uncertainty factoring into the models characterizing resource dynamics, and the idea is to identify the marginal loss of value in one source after accounting for the effect of others. With two sources of uncertainty, this is accomplished by first calculating an average of *EVPI*s for models within groups defined by one of the uncertainty indices, and then subtracting this average from the expected value of perfect information using all the models. The residual after subtraction is the expected value of partial information for the grouping index. *EVPXI* is a partial value of information because it effectively reduces the “total variation” expressed by *EVPI* for the whole model set, by adjusting for “within group variation” associated with differences among models with a common index.

The general patterns for *EVPI* carry over for *EVPXI*. Just as similarities among model value functions tend to reduce the magnitude of *EVPI*[*x*, *q*], so too do similarities in the value functions for models with index *k*′ tend to reduce the magnitude of *EVPI*(*x*, *q*_*k*′′|*k*′_). If these similarities hold across all values of *k*′, then 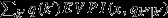 will be small and in consequence *EVPXI*^*K*′^(*x*, *q*) will be large. Essentially, small “within-group variation” in model values results in a large residual value of information that is expressed in *EVPXI*^*K*′^(*x*, *q*). Conversely, relatively large differences in the value functions for models with a common index *k*′ means that *EVPI*(*x*, *q*_*k*′′|*k*′_) will be large, so that *EVPXI*^*K*′^(*x*, *q*) will be small.

We noted above that change in value represented by *EVPI* is relatively small for the pink-footed goose, a result that is consistent with other wildlife applications. There may be a tendency to over-interpret this pattern as suggesting that uncertainty is not an important factor in resource management. However, we take issue with such an interpretation. In fact, *EVPI* indicates how much more value could be gained with decision making if the remainder of the uncertainty facing decision makers could be eliminated. But that in no way devalues the use of information about resource status and processes in decision making. From the above, *EVPI* consists of a comparison of the average of optimal values assuming full process understanding versus the optimal value that is attainable in the face of limited understanding. As such it is effectively a marginal analysis, addressing the marginal value of additional information gathering and assuming an ongoing if imperfect effort to inform decision making. Monitoring is required for the state- and process-based information on which the optimal resource decision making depends, and the question here is whether additional monitoring is justified by the potential increase in value that would be produced. Whether to increase the effort depends on the answer to this question; whether to terminate monitoring altogether does not. It would simply be incorrect to conclude that small *EVPI* means uncertainty is “not a big deal,” or that monitoring information is unneeded.

Finally, it should be emphasized that the value of information, although useful as a measure of the potential change in management performance, does not fully capture the overall value of smart decision making in the face of uncertainty. The framework articulated above for assessing the value of information, involving specified management objectives, a range of potential actions, sources of uncertainty, models that forecast resource changes, and measures of model confidence, provides a platform for comparative assessment among alternative decisions, whether or not decision making is optimal and whether or not the value of information is assessed. The framework helps to motivate and justify postdecision monitoring and assessment as a way to track resource responses and evaluate progress toward objectives. Importantly, it can serve as a mechanism for collaboration and shared decision making, and thereby lower potential contentiousness and conflict among stakeholders. These and other benefits accrue to a structured process for decision assessment and identification. The value of information can certainly contribute to management, but it should be recognized that it is not the only measure of management effectiveness.
